# Successful Endovascular Repair of a Symptomatic Descending Thoracic Aortic Aneurysm in a 90-Year-Old Gentleman: A Case Report

**DOI:** 10.7759/cureus.83940

**Published:** 2025-05-12

**Authors:** Arindam Pande, Arnab Bera, Durlabh Debbarma, Tanmay Banerjee, Rabin Chakraborty

**Affiliations:** 1 Cardiology, Medica Superspecialty Hospital, Kolkata, IND; 2 Chest Medicine, Medica Superspecialty Hospital, Kolkata, IND; 3 Critical Care Medicine, Medica Superspecialty Hospital, Kolkata, IND

**Keywords:** endovascular repair, hemoptysis, high-risk patients, tevar, thoracic aortic aneurysm

## Abstract

Thoracic aortic aneurysms (TAAs) carry high mortality without intervention, and thoracic endovascular aortic repair (TEVAR) has become a preferred minimally invasive option for high-risk patients. We report a 90-year-old male with prostate cancer and type 2 diabetes, who presented with hemoptysis and left-sided chest pain, initially attributed to lobar pneumonia but later diagnosed as a 40 mm saccular descending TAA compressing the left lung on CT angiography. The patient underwent successful TEVAR with complete symptom resolution and was discharged within five days, demonstrating TEVAR's efficacy in fragile elderly patients. This case highlights TEVAR's advantages over open repair, including reduced morbidity and faster recovery, while underscoring the need for further research on long-term outcomes and anatomical limitations in complex cases to refine patient selection and expand applications.

## Introduction

Thoracic aortic aneurysms (TAAs) represent a critical vascular pathology involving pathological dilation of the aortic wall, with an estimated annual incidence of 5-10 per 100,000 population [1]. Among these, type B aneurysms (involving the descending aorta) constitute about one-third of cases. The natural history of untreated TAAs carries significant mortality risk, with reported rates of 11.8% for unruptured cases and catastrophic outcomes (97-100% mortality) following rupture [2]. Given this substantial risk, careful surveillance is mandatory, with timely intervention indicated when rupture risk becomes significant. While conventional open repair remains a definitive treatment, its substantial invasiveness often precludes use in elderly or medically compromised patients. The development of thoracic endovascular aortic repair (TEVAR) has revolutionized management, providing a less invasive option with demonstrably improved perioperative outcomes [3]. For complex anatomical situations involving distal aortic segments, hybrid techniques combining endovascular and open approaches have been developed to simplify procedural demands. Nevertheless, TEVAR application faces technical constraints in cases with challenging vascular anatomy or inadequate proximal/distal sealing zones, angulated necks, mural thrombus, and short landing zones [4].

We report a case of a 90-year-old gentleman with a symptomatic descending TAA who was successfully managed with TEVAR, demonstrating its potential as a safe and effective treatment option for high-risk patients.

## Case presentation

**Figure 1:**
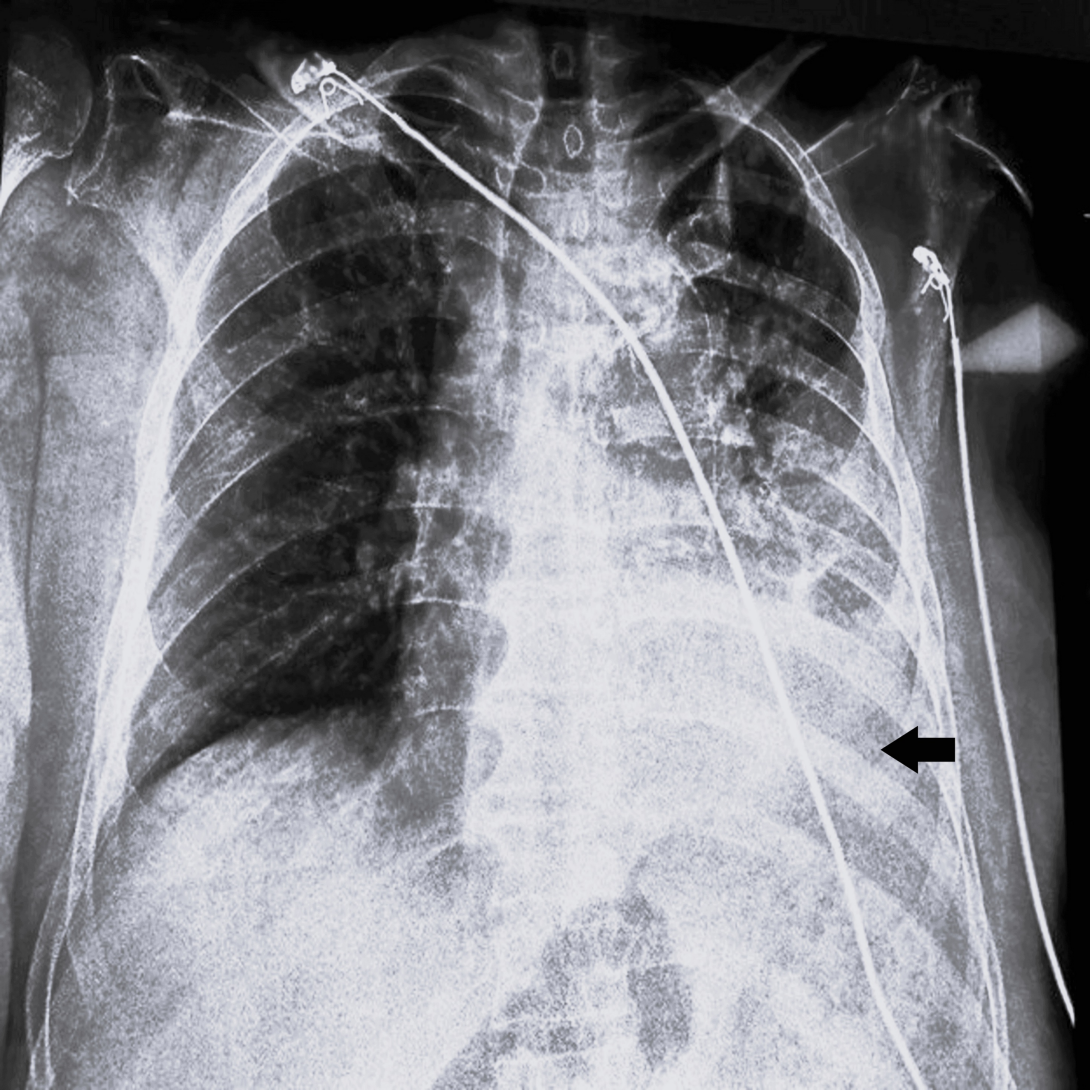
Chest X-ray (posteroanterior view) suggestive of left-sided lobar pneumonia (black arrow pointing toward the segments of consolidation).

A 90-year-old male with a history of prostate cancer (now in remission) and type 2 diabetes mellitus presented with hemoptysis and left-sided chest pain. Initial chest X-ray suggested lobar pneumonia (Figure 1), and he was treated conservatively with intravenous antibiotics and tranexamic acid. Despite treatment, his hemoptysis persisted, and he required high oxygen support (4 liters through a mask).

**Figure 2:**
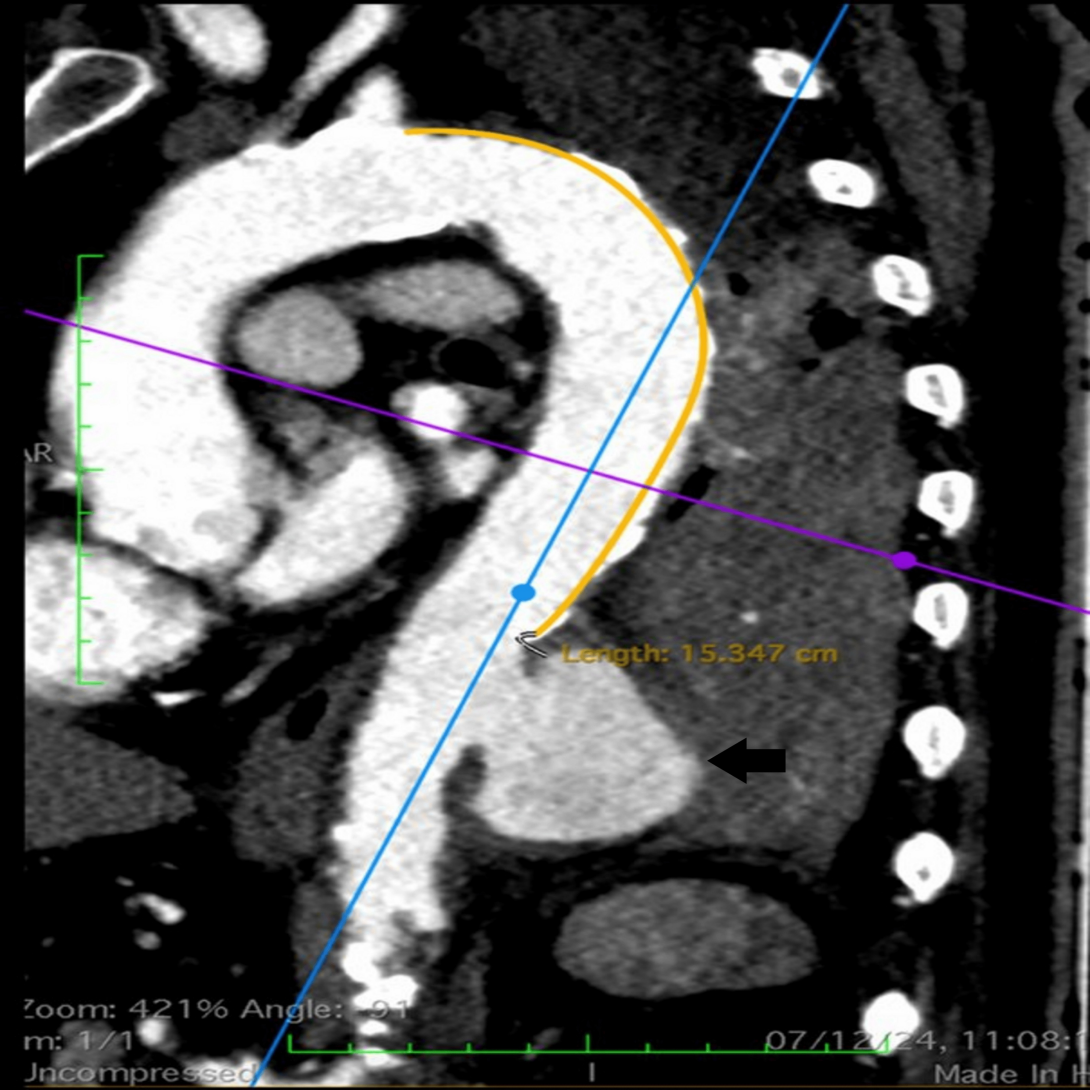
CT aortogram showing a 40 mm saccular aneurysm in the descending thoracic aorta, adjacent to the left lung parenchyma, which was compressing the left lung, causing uncontrollable bloody sputum (black arrow pointing to the saccular aneurysm).

Failure of conventional treatment prompted a contrast-enhanced computed tomography (CECT) scan of the thorax, which revealed scattered fibrosis, passive segmental collapse, and consolidation in the left lower lobe. Additionally, a 40 mm saccular aneurysm was identified in the descending thoracic aorta, compressing the left lung parenchyma. A CT aortogram confirmed the presence of a mural thrombus within the aneurysm, which had enlarged to such an extent that it resembled a "new baby heart" within the thoracic cavity (Figure 2).

**Figure 3:**
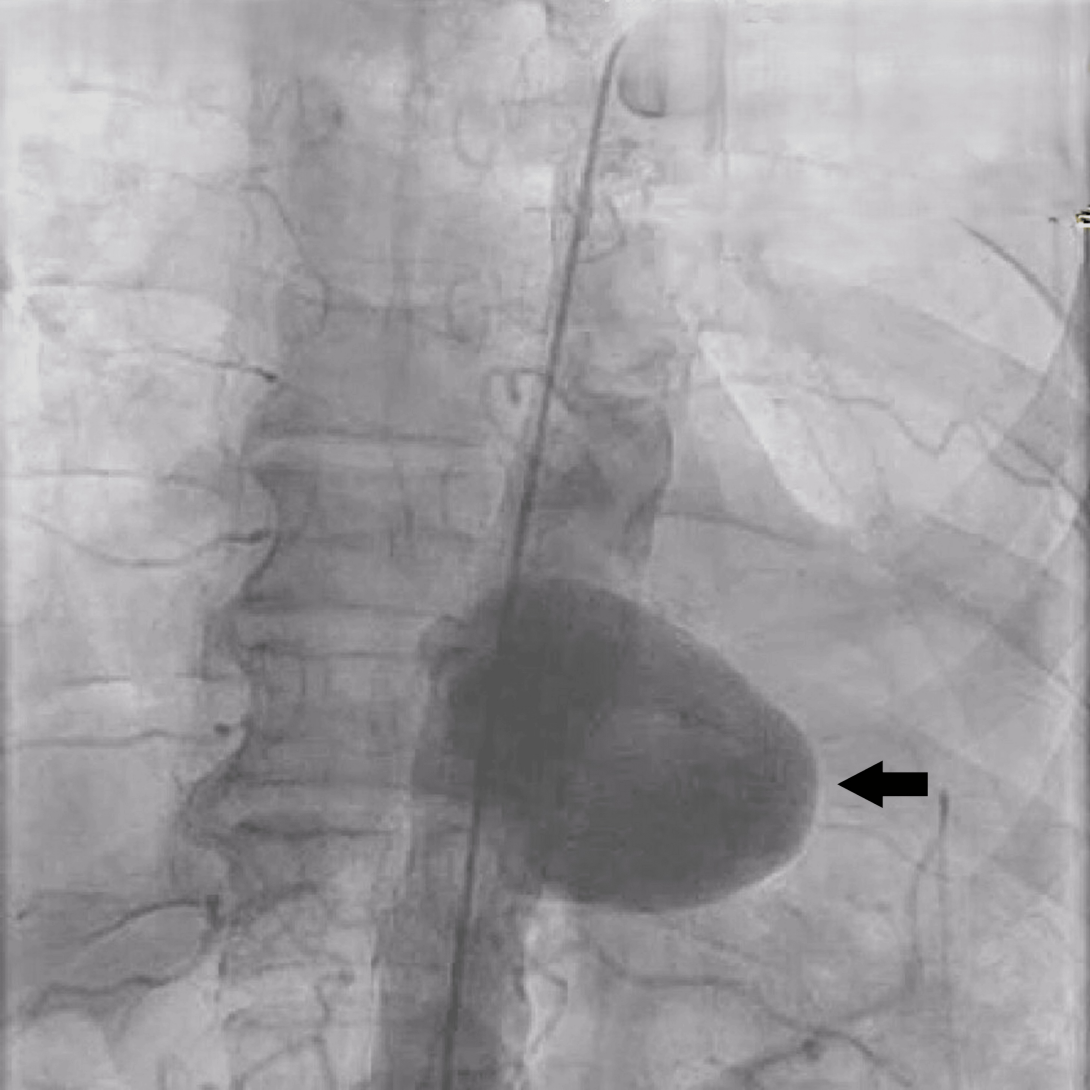
Aortogram showing the saccular aneurysm (black arrow) in the descending thoracic aorta.

**Figure 4:**
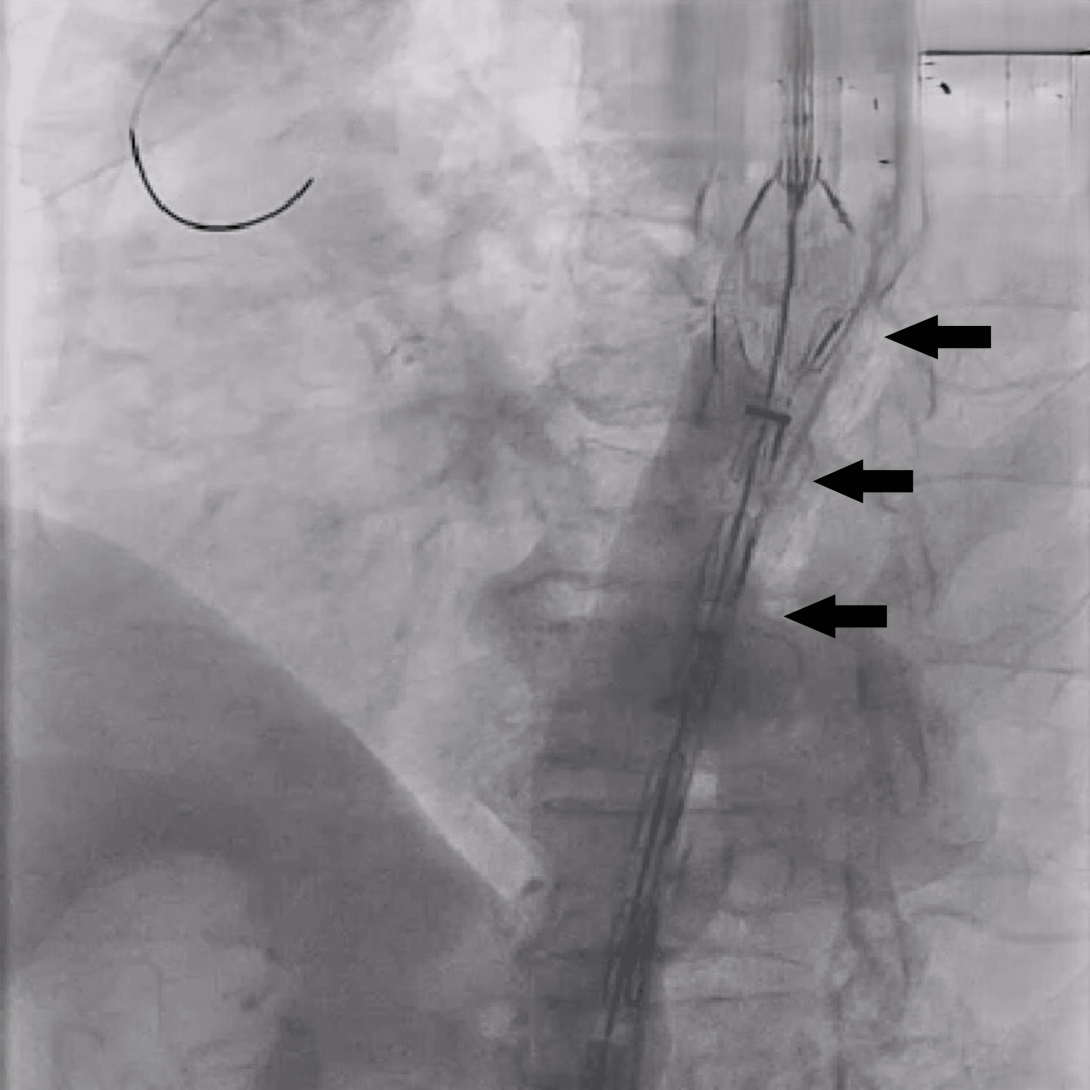
Deployment of the 120/30 mm stent graft (Lifetech Scientific) in the aneurysm part, pointed by black arrows.

**Figure 5:**
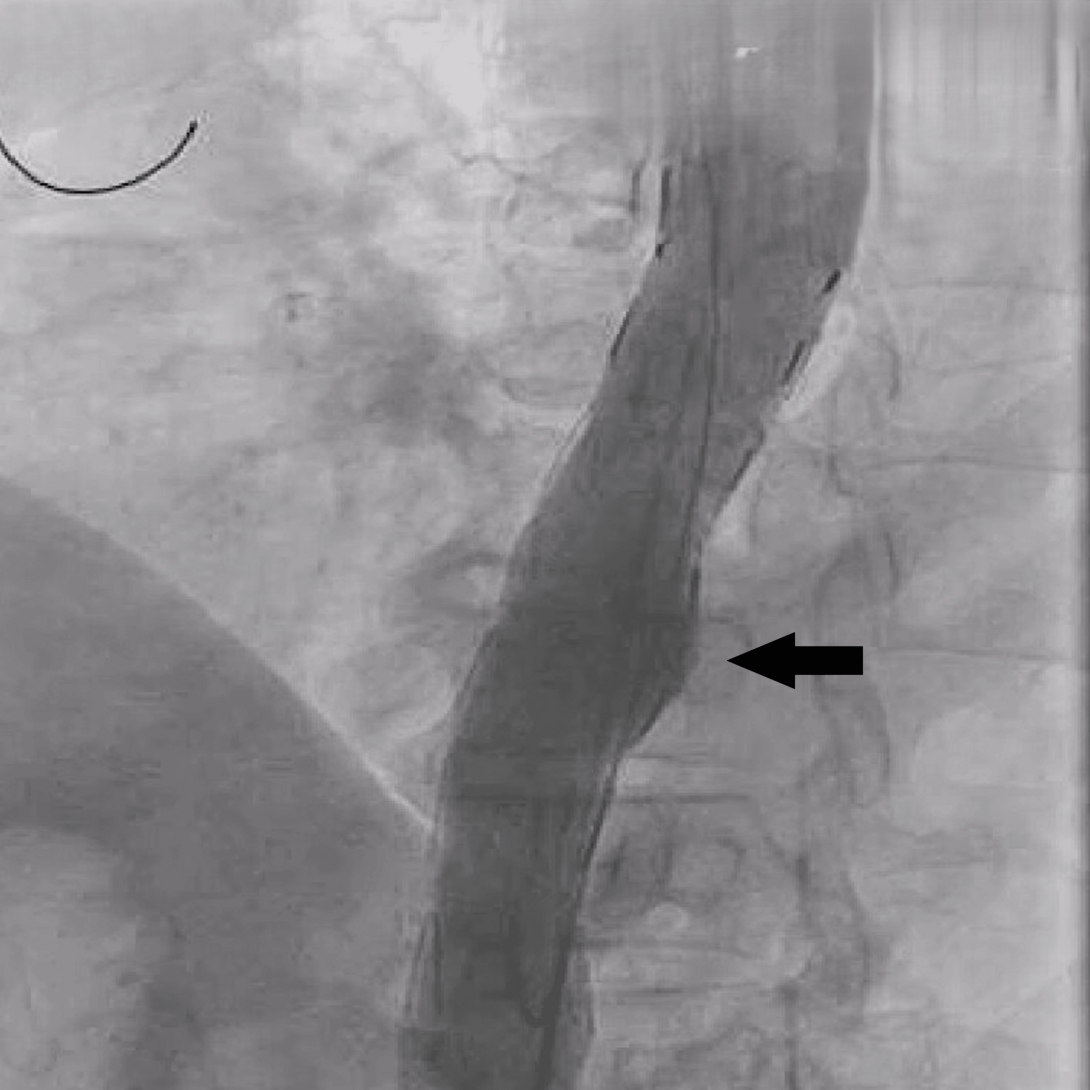
Final aortogram after the successful stent deployment. Black arrow pointing to the complete isolation of the aneurysm cavity without any residual leak.

**Figure 6:**
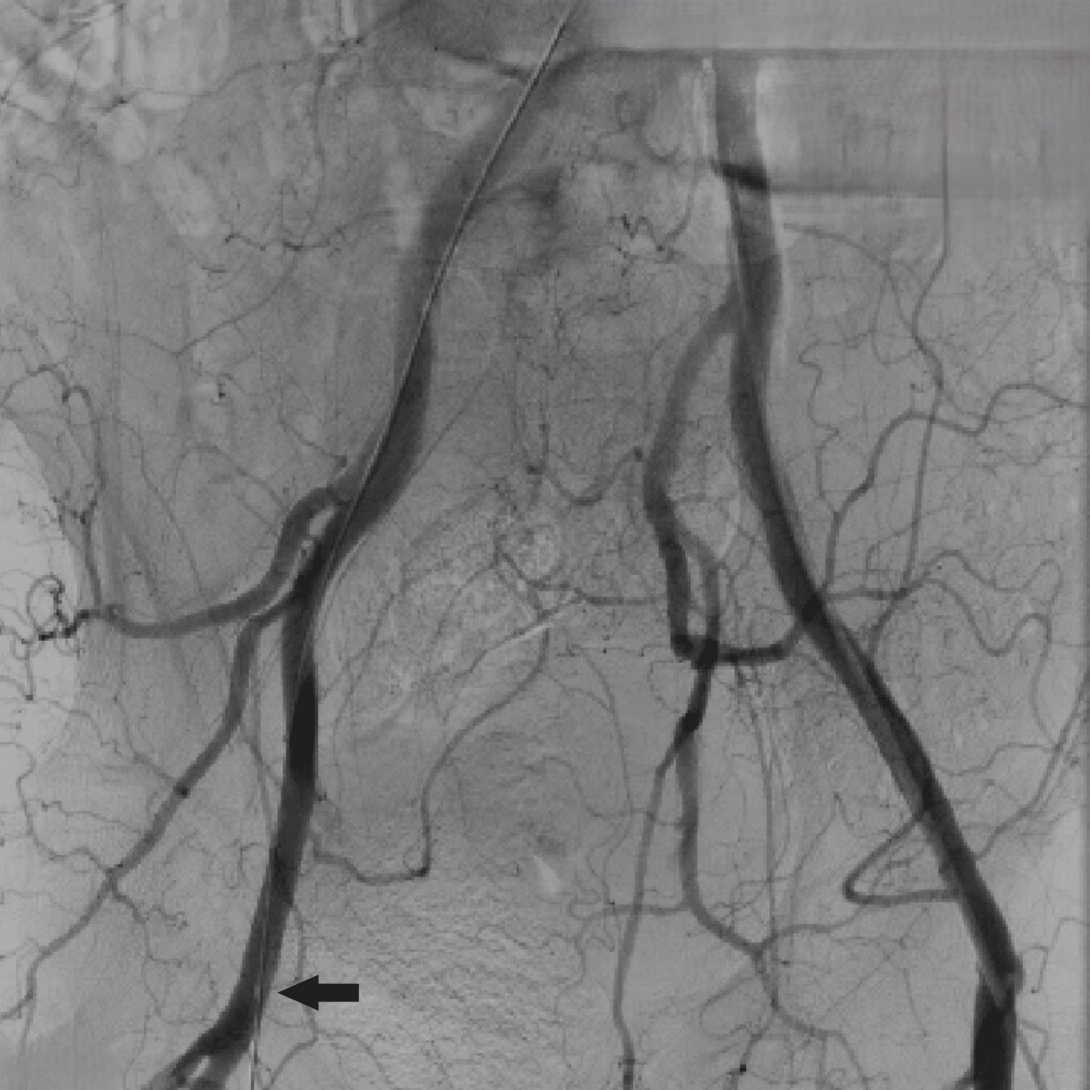
Final aortogram showing 20 F femoral access closed by Manta Device (Teleflex Medical Pvt. Ltd.), pointed by a black arrow.

Given the patient’s age, comorbidities, and high surgical risk, TEVAR was deemed the most appropriate intervention. An aortogram confirmed the position of the aneurysm (Figure 3). A 120/30 mm stent graft (Lifetech Scientific, Shenzhen, China) was deployed successfully under conscious sedation (Figure 4), with no procedural complications. Post-procedure imaging confirmed complete exclusion of the aneurysm (Figure 5), and the femoral access site was closed using a Manta device (Teleflex Medical Pvt. Ltd., Thirubuvanai, India) (Figure 6).

**Figure 7:**
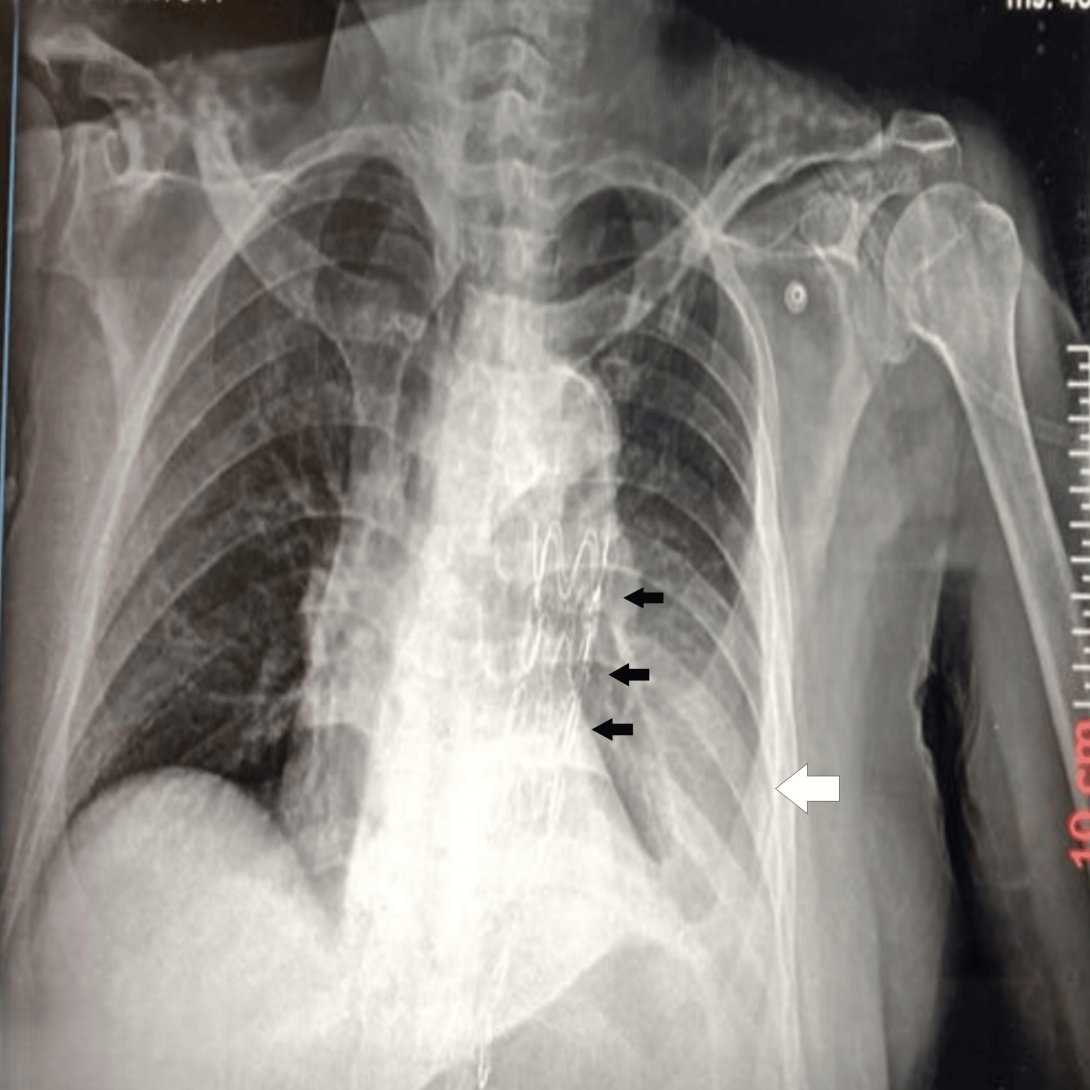
Repeat chest X-ray after 48 hours of procedure showing improvement of lung opacities (pointed by a white arrow) and stent graft in descending thoracic aorta (pointed by black arrows).

The patient’s hemoptysis resolved gradually, and he was discharged after five days in stable condition. A follow-up chest X-ray at 48 hours post procedure showed no complications and resolution of lung opacities (Figure 7), and he remained asymptomatic at the 14-day outpatient visit. A longer-term follow-up with imaging is planned or has been done, as aneurysm-related complications can be delayed.

## Discussion

TAAs most commonly develop due to cystic medial necrosis, a degenerative condition that breaks down the elastic and muscular fibers in the aortic wall’s middle layer (tunica media). This structural weakening, combined with the constant stress of blood flow, leads to progressive dilation. Importantly, true aneurysms must be distinguished from false aneurysms, essentially contained hematomas often caused by trauma or surgical interventions, and mycotic aneurysms, which are diagnosed through clinical criteria such as systemic infection, positive cultures, or distinctive imaging features [5,6]. Additionally, aneurysms can form as a complication of acute aortic dissection.

TAAs predominantly occur in the ascending aorta, though they also frequently affect the descending aorta and aortic arch. Most patients are diagnosed between 60 and 70 years of age [5,7,8]. To our knowledge, there is no reported case with a positive outcome in a nonagenarian in the literature. Interestingly, studies show that women with degenerative TAAs experience faster aneurysm growth than men, even after accounting for differences in body size or other health factors [8].

Clinically, TAAs often present without symptoms and are discovered incidentally during imaging for unrelated conditions. When symptoms do occur, they may include a diastolic murmur or, in rare cases, signs of heart failure. Larger aneurysms can compress nearby structures like the trachea or bronchi, leading to coughing, shortness of breath, wheezing, chest pain, or even hemoptysis [5,9,10]. Severe complications, though uncommon, include aortoesophageal fistulas and rupture, the latter often heralded by sudden, excruciating pain in the chest, back, or abdomen. Chiari’s triad, comprising (a) midthoracic pain, (b) a sentinel bleed, and (c) delayed massive hemorrhage, characterizes this catastrophic event. Without emergency surgery, rupture is invariably fatal; even with intervention, mortality rates remain high (30-80%) [11,12].

Diagnosis relies on contrast-enhanced CT scans or magnetic resonance angiography, which provide detailed imaging of the aorta [5,9]. Many TAAs are detected incidentally during evaluations for unrelated issues, such as in this patient, who was diagnosed while being assessed for persistent hemoptysis [5,9,10,13].

Management depends on aneurysm size and patient-specific factors. Smaller aneurysms (under 6 cm) are typically managed medically unless they cause symptoms or are accompanied by high-risk conditions. Propranolol has been shown to slow aortic dilation and reduce complications compared to other treatments [5,10]. Surgical intervention is generally recommended when the aneurysm reaches 5.5 cm in the ascending aorta or 6.5 cm in the descending aorta, or if it grows more than 1 cm per year.

While open surgical repair remains an option, TEVAR has become a preferred, less invasive alternative, particularly for high-risk or elderly patients. TEVAR can be performed under regional anesthesia (RA), avoiding intubation and reducing postoperative complications, or general anesthesia (GA) for complex cases requiring precise stent placement or prolonged procedures [14]. Despite its advantages, challenges such as ensuring adequate landing zones for stent grafts and long-term durability data remain areas of ongoing research.

Postoperative quality of life (QoL) is an understudied aspect of TAA repair, especially in elderly or frail patients who may face prolonged recovery or loss of independence. Since many TAAs are asymptomatic before surgery, weighing the risks and benefits of intervention is crucial. Currently, no universal treatment algorithm exists, emphasizing the need for personalized decision-making based on aneurysm characteristics, patient health, and anticipated outcomes. Those with rapidly expanding or large aneurysms are prioritized for surgery, though this complicates our understanding of the disease’s natural progression. Advances in molecular imaging and targeted therapies, however, offer hope for improved future management.

This case highlights the potential of TEVAR to improve outcomes in high-risk patients, as demonstrated by the successful treatment of a 90-year-old individual. However, further research is needed to optimize patient selection, refine techniques, and evaluate long-term stent performance. A short follow-up of the index case and a lack of published data in the very elderly population are important limitations in the proposed approach.

## Conclusions

TEVAR has emerged as a safe and effective therapeutic alternative for symptomatic descending TAAs, particularly in high-risk surgical candidates. The presented case highlights TEVAR's capability to deliver favorable clinical outcomes while minimizing procedural morbidity. However, comprehensive multicenter studies and extended follow-up data remain imperative to fully elucidate its long-term efficacy and establish optimal applications for complex aortic pathologies.
